# Invasive Airway Breathing Circulation Disability: A Comprehensive Course for Emergency Physicians

**DOI:** 10.7759/cureus.91465

**Published:** 2025-09-02

**Authors:** Upendra Hansda, Madhumita Patnaik, Sangeeta Sahoo, Pankaj Kumar, Chinmaya Dash, Saroj K Sahoo, Manisha R Gaikwad, Sadananda Barik

**Affiliations:** 1 Trauma and Emergency, All India Institute of Medical Sciences, Bhubaneswar, IND; 2 Anatomy, All India Institute of Medical Sciences, Bhubaneswar, IND; 3 General Surgery, All India Institute of Medical Sciences, Bhubaneswar, IND; 4 Cardiology, All India Institute of Medical Sciences, Bhubaneswar, IND

**Keywords:** airway procedures, central line insertion, emergency chest drain, emergency pericardiocentesis, external ventricular drain (evd)

## Abstract

Background

The invasive airway, breathing, circulation, and disability workshop was aimed at enhancing the competency of emergency physicians in life-saving procedures, crucial in the emergency room during resuscitation.

Objectives

The primary objective was to assess the improvement in the participants' knowledge before and after the workshop. The secondary objective was to assess the confidence level of the participants based on their feedback.

Method

A pre-test and post-test were conducted for the participants. Initial didactic lectures followed by hands-on skills practice were carried out. The participants were allocated into eight groups. Each group had to rotate every half an hour so that each participant could get adequate time for practice on the cadaver or manikin. A faculty/facilitator expert in the respective field supervised each skill station. Structured feedback was obtained from the participants after the workshop.

Results

Thirty-seven participants attended the workshop. The median pre-test score was 8 (IQR 6-9), and the post-test score was 13 (IQR 12-14). There was a significant increase in the knowledge level of the participants (p <0.001). The participants’ confidence in performing fibreoptic intubation, cricothyroidotomy, pericardiocentesis, chest tube insertion, central line, and external ventricular drain was increased significantly (p<0.001).

Conclusion

This teaching and training program on invasive procedural skills has increased the knowledge and confidence of the participants.

## Introduction

Invasive life-saving procedures form the backbone of effective resuscitation in emergency settings. When executed timely and accurately, they can be the difference between life and death. However, improper execution due to a lack of hands-on experience or confidence can have fatal consequences. To bridge this critical gap, structured skills lab training programs are increasingly recognized as essential components of medical education, especially for residents in emergency medicine [1]. Such programs allow participants to gain both cognitive understanding and psychomotor proficiency in a safe, controlled, and psychologically secure environment [2].

The “i-ABCD for emergency physicians” workshop (short for invasive airway, breathing, circulation, and disability) was designed to enhance the procedural competency of emergency physicians by providing immersive, hands-on training in life-saving techniques vital during resuscitation [3]. In India, emergency medicine is still evolving as a formal specialty, and structured simulation-based training programs remain limited. Hence, there is an urgent need to standardize procedural training that can boost both the knowledge and the confidence of healthcare providers in high-stakes environments such as the emergency room.

Multiple studies have emphasized the value of cadaver-based and manikin-based simulation in skill acquisition [4]. Simulation provides a realistic yet risk-free setting to hone technical abilities and decision-making skills. Prior research has shown that cadaveric training improves not only anatomical understanding but also surgical dexterity and confidence, especially in rare or complex procedures [5]. For instance, in Japan, emergency and critical care medicine ranked as the second-highest area utilizing cadaver surgical training after orthopedics, underlining its perceived importance [6]. Furthermore, over 90% of cadaveric training programs in Japan were intended for educational purposes, particularly in fields like surgery and emergency medicine [7].

Despite these international precedents, there remains a scarcity of data in the Indian context evaluating the effectiveness of such interventions in enhancing clinical competence. Most training programs lack a formal assessment of participant outcomes in terms of knowledge improvement or confidence gain. The “i-ABCD” workshop was developed to address this gap.

This study was therefore conducted with the primary objective of evaluating the improvement in participants’ knowledge before and after the “i-ABCD” workshop. The secondary objective was to assess the change in participants’ confidence levels based on structured post-training feedback. By combining didactic sessions with rotational hands-on practice on cadavers and manikins, each supervised by a faculty expert (master’s degree qualified in the disciplines of Anaesthesiology, Cardiology, Pulmonary Medicine, General Surgery, and Neurosurgery), this training aimed to create a comprehensive, immersive learning experience.

The insights gained from this study are expected to support the integration of such simulation-based, skill-intensive modules into the standard residency curriculum, ensuring that emergency physicians are well-equipped to perform critical invasive procedures with both competence and confidence.

## Materials and methods

This study was conducted at a tertiary care teaching institute in India after the ethical clearance (IEC No -T/IM-NF/T&EM/23/148 dated 5 Feb 2024). This study aimed to assess knowledge improvement and confidence enhancement following a structured, simulation-based invasive procedure workshop titled “i-ABCD for emergency physicians”.

Study design and participants

This was a retrospective study. Participants included the faculty with less than one year of independent clinical experience in emergency care and postgraduate trainees in emergency medicine, anaesthesiology, and surgery. 

Pre-workshop assessment

Prior to the commencement of the workshop, all participants completed a validated, structured pre-test questionnaire designed to assess baseline knowledge of life-saving invasive procedures. The questionnaire consisted of 15 multiple-choice questions, each with a single best answer, covering theoretical and practical aspects of airway, breathing, circulation, and neurological access procedures.

Workshop structure

The workshop was conducted in two phases: an interactive lecture session and a hands-on skill station. The interactive lecture sessions provided theoretical insights into advanced invasive procedures, including direct laryngoscopy, fiberoptic intubation, cricothyrotomy, chest tube insertion, central venous access, needle thoracostomy, pericardiocentesis, intraosseous access, and external ventricular drain (EVD) placement. In hands-on skills, eight skill stations were designed for hands-on practice, with procedures assigned based on anatomical fidelity and equipment availability. The skills for direct laryngoscopy (Airway Larry Trainer, Nasco Healthcare Inc., New York, USA), fiberoptic intubation (Ambu aScope 4 Regular, Penang, Malaysia), central line insertion (Gen II Ultrasound Central Line Training Model, GT Simulators by Global Technologies, Davie, FL, USA), needle thoracostomy (Tension Pneumothorax Simulator, Simulaids, Leicestershire, UK), and intraosseous access (Arrow™ EZ-IO™ Needle, Teleflex Incorporated, Pennsylvania, USA) were taught using a manikin. The cricothyrotomy, intercostal chest drain insertion, and EVD placement were taught using cadavers. Ultrasonography probe orientation was demonstrated on human volunteers, and needle orientation was practiced using a Blue Phantom simulator (Regional Anesthesia Ultrasound Training Block, GT Simulators by Global Technologies, Davie, FL, USA).

Participants were divided into eight groups, each comprising five members. Groups rotated through all eight stations, spending 30 minutes per station. Each station was supervised by two expert facilitators, ensuring that every participant performed each procedure under direct observation and received personalized guidance.

Post-workshop assessment

Immediately following the completion of hands-on sessions, participants were reassessed using the same questionnaire as the pre-test. Additionally, a structured feedback form using a Likert scale was used to evaluate self-reported confidence levels (1- afraid of doing, 2- not sure, 3- can do under supervision, and 4- can do independently) in performing each procedure, perceived effectiveness of the training, and satisfaction (1- very poor, 2- poor, 3- fair, 4- very good, 5- excellent) with the course structure and delivery.

Statistical analysis

Descriptive statistics (mean, standard deviation, frequencies) were used to summarize participant demographics, pre- and post-test scores, and feedback responses. Paired t-test (for normally distributed data) or Wilcoxon signed-rank test (for non-parametric data) was used to compare pre-test and post-test scores, assessing knowledge improvement. Effect size (Cohen’s d) was calculated to quantify the magnitude of knowledge gain. Median and interquartile ranges are reported wherever necessary. Internal consistency of the questionnaire was assessed using Cronbach’s alpha. P-values < 0.05 were considered statistically significant. All statistical analyses were performed using IBM SPSS Statistics for Windows, Version 20 (Released 2011; IBM Corp., Armonk, New York, United States).

## Results

A total of 41 participants attended the "i-ABCD" workshop. However, data from 37 participants were included in the final analysis, as four attendees arrived late and did not complete the pre-test. Among the analyzed participants, 21 (56.7%, n=37) were emergency medicine resident trainees, 14 (37.8%, n=37) were from anesthesiology, and two (5.4%, n=37) were from surgery. 

Knowledge improvement

The pre-test median score was 8 (interquartile range (IQR): 6-9), which significantly improved to 13 (IQR: 12-14) in the post-test. This difference was statistically significant (p < 0.001, Wilcoxon signed-rank test), demonstrating a substantial improvement in participants’ theoretical knowledge following the workshop. The effect size of 0.87 indicated a strong practical significance of the intervention.

Participant feedback

**Figure 1:**
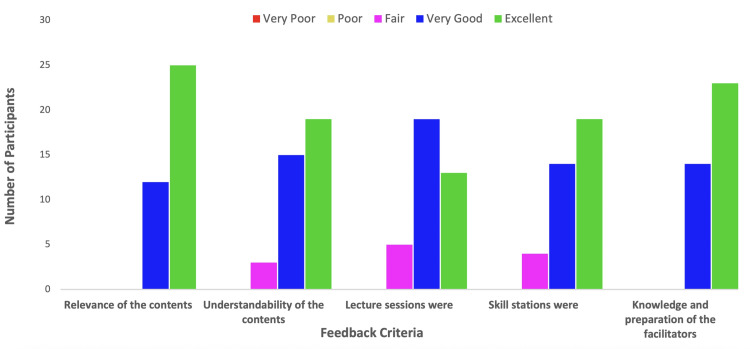
Feedback from the participants

**Figure 2:**
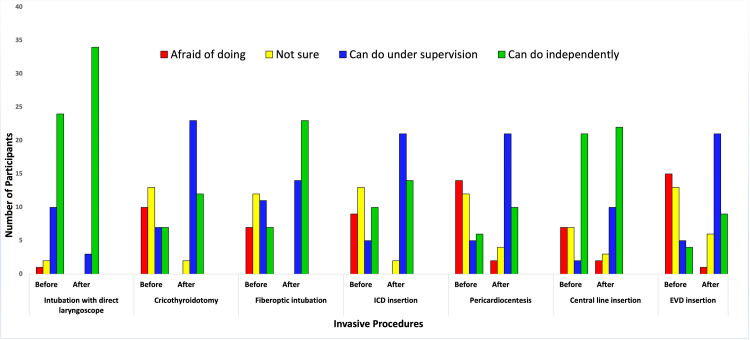
Confidence to perform procedures before and after the workshop EVD: External ventricular drain; ICD: Intercostal chest drain

There was a high level of satisfaction amongst the participants, which was collected using structured feedback. A majority of them rated the relevance, clarity of lectures, quality of skills teaching, and facilitators’ expertise as “very good” to “excellent” (Figure 1). The participants’ self-reported confidence levels in performing invasive procedures significantly increased across all skill domains (Table 1, Figure 2), aligning with the workshop’s secondary objective.

**Table 1:** Confidence to perform the invasive procedures before and after the workshop *Wilcoxon Signed Rank. EVD: External ventricular drain; IQR: Interquartile range; ICD: Intercostal chest drain

	Median (IQR): Before the Workshop	Median (IQR): After the Workshop	p-value*
Intubation with a laryngoscope	4 (3-4)	4 (4-4)	<0.001
Fibreoptic intubation	2 (2-3)	4 (3-4)	<0.001
Cricothyroidotomy	2 (1-3)	3 (3-4)	<0.001
ICD insertion	2 (2-4)	3 (3-4)	<0.001
Pericardiocentesis	2 (1-3)	3 (3-4)	<0.001
Central line insertion	4 (2-4)	4 (3-4)	<0.05
EVD insertion	2 (1-2)	3 (3-3)	<0.001
Self-reflection	3 (3-4)	4 (4-5)	<0.001

Qualitative feedback

Fifty-seven percent (n=37) of the participants highlighted the hands-on skill stations, 24% (n=37) appreciated the content and structure of the course, 11% (n=37) valued the knowledge and teaching style of the facilitators, and eight percent (n=37) emphasized the opportunity to practice on cadavers as a unique learning experience. Notably, 100% of participants expressed that they would recommend this workshop to their peers and considered it a valuable component of emergency medicine training.

## Discussion

This study evaluated the impact of a structured, advanced invasive procedure workshop (i-ABCD) on the knowledge and confidence of the participants. The results demonstrated a statistically significant improvement in both domains, affirming the value of simulation-based procedural training in acute care settings.

The i-ABCD workshop was designed to enhance core procedural competencies critical for patient resuscitation in the emergency department. The course covered essential invasive interventions such as direct and fiberoptic intubation, cricothyrotomy, central venous catheterization, needle thoracostomy, intraosseous access, intercostal chest drain insertion, pericardiocentesis, and EVD placement. The combination of didactic teaching and hands-on skills, delivered through cadaveric and manikin-based simulation, provided participants with both conceptual understanding and psychomotor reinforcement.

This format is consistent with educational best practices. Procedural skills, particularly those involving surgical and invasive techniques, are most effectively learned through deliberate practice that combines cognitive input and kinesthetic rehearsal. Studies emphasize that without a structured approach informed by motor learning theories, procedural training may not lead to durable clinical competence [8]. Our program addressed this need by integrating theoretical instruction prior to supervised practice.

The observed increase in median test scores from 8 (IQR 6-9) in the pre-test to 13 (IQR 12-14) in the post-test confirms a quantifiable knowledge gain. Similarly, feedback responses demonstrated a marked improvement in participants’ confidence across all procedures taught, as rated on a four-point Likert scale. These findings are consistent with existing literature. For instance, Lee et al. found that structured cadaveric and simulation-based programs significantly enhanced perceived training adequacy, opportunity for practice, and clarity of instruction [9].

Our course content extended beyond that of standard trauma courses such as Advanced Trauma Life Support (ATLS), which focuses primarily on core emergency procedures like cricothyrotomy, needle decompression, tube thoracostomy, central venous access, and pericardiocentesis [10]. In addition to these, the i-ABCD workshop included fiberoptic intubation and EVD placement, addressing procedures more commonly encountered in neurocritical and advanced airway management scenarios. The inclusion of such procedures responds to the evolving demands of emergency medicine, especially in academic and tertiary care settings.

Prior research supports the inclusion of cadaveric training for building technical competence. Training on human cadavers has improved the confidence and skill levels of general surgery residents, undergraduate medical students, and paramedics [11-13]. Our study echoes these findings in the context of emergency medicine training. Although we used formalin-embalmed cadavers, which are less realistic than fresh-frozen or Thiel-embalmed models, they offered adequate anatomical fidelity for procedural learning at a fraction of the cost [14]. Participants reported high satisfaction with the cadaveric experience, consistent with other studies that found increased satisfaction and confidence in cadaver or animal-model-based training [15].

Emerging literature has explored various simulation models and teaching methods. For example, the Pecha Kucha method and part-task training have shown effectiveness in fiberoptic intubation education [16]. Similarly, 3D-printed trainers have been used for the realistic simulation of bleeding cricothyrotomy, while goat cadavers have been successfully employed in pediatric emergency training in resource-limited settings [13,17]. These innovations reinforce the idea that a well-designed educational experience, regardless of the simulation model, can positively influence procedural learning.

Feedback from our participants indicated that the hands-on skills stations were the most appreciated part of the course (57%, n=37), followed by the course content (24%, n=37), facilitator expertise (11%, n=37), and the opportunity to practice on cadavers (8%, n=37). These findings mirror the work of Hsu et al., who found that hybrid simulation models can enhance student engagement and perceived preparedness [18]. Our results also align with findings from battlefield simulation training programs, which reported increased confidence across multiple invasive procedures following high-fidelity cadaveric training [19].

The structured delivery of our course was consistent with the CACTUS (ChAracteristics of cadaver training and sUrgical studies) guidelines, ensuring that the training included clear objectives, expert facilitation, and sufficient hands-on time [20]. Each skill station was staffed with two facilitators, allowing for detailed supervision and real-time feedback. The rotating group format ensured that all participants had equitable access to procedural practice.

Despite the strengths of our training model, the study has certain limitations. It was conducted at a single center with a relatively small sample size (n=37), limiting generalizability. Although the participants' knowledge and confidence improved significantly, the study did not assess long-term retention or real-world clinical performance. Moreover, the feedback was subjective, based on self-reported measures rather than objective assessment tools or OSCE-based evaluations. Future studies could incorporate performance-based assessments and follow-up evaluations to determine knowledge retention and transferability to clinical practice.

## Conclusions

The i-ABCD course demonstrated that a structured, simulation-based training program can significantly enhance knowledge and procedural confidence in emergency physicians. The inclusion of advanced, life-saving invasive techniques in a controlled and supervised environment provides a safe and effective platform for skill acquisition. Given its feasibility, adaptability, and impact, the i-ABCD model can be integrated into residency programs and continuing medical education curricula to support skill development and improve patient care outcomes in emergency settings.

## Figures and Tables

**Figure 3:**
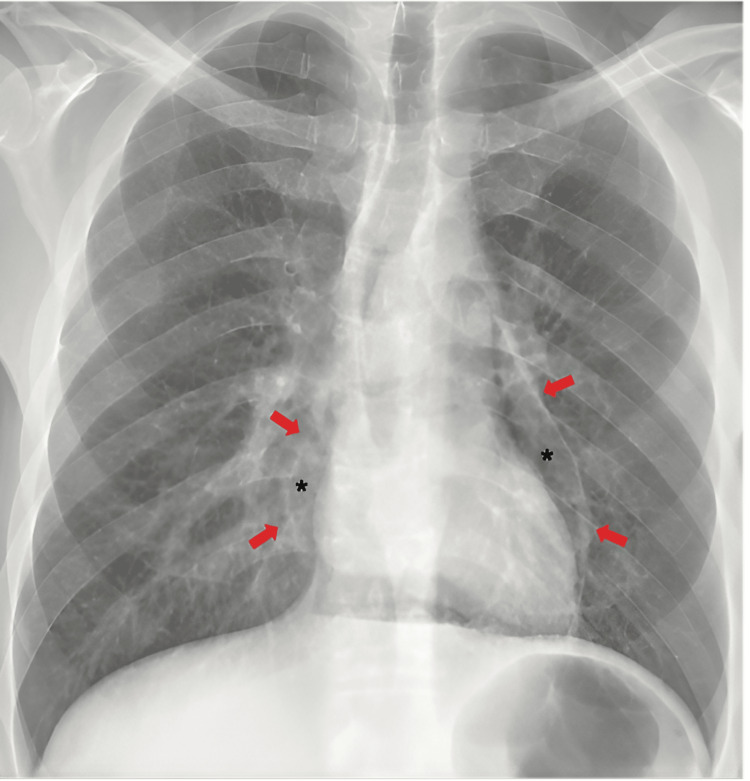
Chest X-ray for question no 1.

## Appendices

Pre- and Post-test questionnaire

1. A 48-year-old man presented to the emergency department with acute dyspnea. At presentation, his blood pressure was 70/50 mmHg, heart rate- 130 beats/min, and SpO2 of 88%. Physical examination showed muffled heart sounds, clear lungs, and distention of the jugular veins. An echocardiogram showed a large circumferential pericardial effusion. Echo-guided pericardiocentesis, under local anesthesia, through the sub-xiphoid approach was done. Drainage of 1000 mL of transudate resulted in rapid clinical improvement. Post-drainage chest X-ray is shown in Figure 3. What is the diagnosis? a. Pericardial effusion recurrence, b. Aortic dissection, c. Ventricular rupture or d. Pneumopericardium.

2. Which of the following is the best method to manage tension pneumothorax?

a. Immediate ICD placement

b. Chest X-ray to confirm tension pneumothorax, followed by needle thoracostomy.

c. Needle thoracostomy followed by X-ray of the chest.

d. Needle thoracostomy followed by ICD insertion.

3. A 24-year-old man presented with shortness of breath after an assault with a knife to the chest. On examination, his SpO2 was 80% on room air, respiratory rate was 34/min, and blood pressure was 82/36 mm Hg. He has a wound on the left side of the chest with a suckling sound. The air entry was reduced on the left side with hyper-resonance on percussion. What is the best way to manage the patient?

a. Suturing of the wound followed by the ICD placement.

b. ICD insertion through the same wound.

c. Three-sided occlusive dressing followed by ICD placement.

d. Needle thoracostomy 

4. A 20-year-old man with severe maxillofacial trauma and head injury is brought to the Emergency Department in an unconscious state. What is the most appropriate method of endotracheal intubation? 

a. Intubation by fiberoptic scope

b. Intubation by direct laryngoscopy

c. Intubation by video laryngoscopy 

d. Nasotracheal intubation

5. A curved laryngoscope blade is designed so that the tip of the blade is inserted at

a. Epiglottis

b. Tonsillar fossa

c. Vallecula

d. Just above the vocal cords

6. The intercostal nerves lie between the

a. External and internal intercostals

b. Innermost intercostals and endothoracic fascia

c. Internal intercostal and innermost intercostal

d. Endothoracic fascia and parietal pleura

7. The fibrous pericardium is innervated by

a. Vagus nerve

b. Phrenic nerve

c. T1 -T5 sympathetic nerves

d. Intercostal nerves

8. When managing an unconscious patient who is making labored and noisy respirations, the emergency physician must first:

a. Commence positive pressure ventilation with a bag-mask device immediately

b. Immediately perform endotracheal intubation

c. Perform a “finger sweep” to clear the oropharynx of foreign material

d. Perform a simple maneuver, such as a head tilt-chin lift, to open the airway

9. The correct size mask to use with the bag-mask ventilation

a. Should cover the mouth and seal against the nose to avoid air leaks during ventilation

b. Should cover the mouth such that oxygen-rich air is able to pass through the nose unobstructed during ventilation

c. Should seal against the nose if a nasopharyngeal airway is used, and seal against the mouth if an oropharyngeal airway is being used

d. Should cover the mouth and nose and create a tight seal to avoid air leaks during positive pressure ventilation

10. The best method to confirm the correct placement of a central venous catheter is

a. Chest X-ray

b. Measurement of the pH of the blood sample drawn from the catheter

c. Measurement of pressure in the catheter using a pressure transducer

d. Aspiration of dark red blood from all the lumens of the catheter 

11. In infants, intraosseous access should avoid which part of the bone?

a. Metaphysis

b. Diaphysis

c. Epiphysis

d. Mid bone

12. The external ventricular drain is to be done on

a. Ipsilateral to pupillary dilation

b. Contralateral to pupillary dilation

c. Ipsilateral to motor paresis

d. Any side

13. In emergency situations, the best way to identify the cricothyroid membrane is 

a. Using ultrasound

b. Laryngeal handshake technique

c. By anatomical landmark 

d. Using a fiber-optic scope

14. Initial skin incision in case of a nonpalpable cricothyroid membrane is

a. 10 cm horizontal

b. 8 cm horizontal

c. 10 cm vertical

d. 8 cm vertical

15. All of the following may be seen in patients of cardiac tamponade except:

a. Kussmaul’s sign

b. Pulsus paradoxus

c. Electrical alternans

d. RV diastolic collapse in 2D echo

Feedback form

1.     Relevance of the contents of the workshop

a.     Very poor

b.     Poor

c.      Fair

d.     Very good

e.     Excellent

2.     Understandability of the contents of the workshop

a.     Very poor

b.     Poor

c.      Fair

d.     Very good

e.     Excellent

3.     Lecture sessions of the workshop were

a.     Very poor

b.     Poor

c.      Fair

d.     Very good

e.     Excellent

4.     Skill stations were

a.     Very poor

b.     Poor

c.      Fair

d.     Very good

e.     Excellent

5.     Knowledge and preparation of the facilitators

a.     Very poor

b.     Poor

c.      Fair

d.     Very good

e.     Excellent

6.     What was your reflection (learning chronicle, skill, and ability) BEFORE the workshop?

a.     Very poor

b.     Poor

c.      Fair

d.     Very good

e.     Excellent

7.     What was your reflection (learning chronicle, skill, and ability) AFTER the workshop?

a.     Very poor

b.     Poor

c.      Fair

d.     Very good

e.     Excellent

8.     Your confidence to perform intubation with a laryngoscope (BEFORE attending the workshop)

a.     Afraid of doing

b.     Not sure

c.      Can do under supervision

d.     Can do independently

9.     Your confidence to perform intubation with a laryngoscope (AFTER attending the workshop)

a.     Afraid of doing

b.     Not sure

c.      Can do under supervision

d.     Can do independently

10.  Your confidence to perform cricothyroidotomy (BEFORE attending the workshop)

a.     Afraid of doing

b.     Not sure

c.      Can do under supervision

d.     Can do independently

11.  Your confidence to perform cricothyroidotomy (AFTER attending the workshop)

a.     Afraid of doing

b.     Not sure

c.      Can do under supervision

d.     Can do independently

12.  Your confidence to perform fibreoptic intubation (BEFORE attending the workshop)

a.     Afraid of doing

b.     Not sure

c.      Can do under supervision

d.     Can do independently

13.  Your confidence to perform fiber-optic intubation (AFTER attending the workshop)

a.     Afraid of doing

b.     Not sure

c.      Can do under supervision

d.     Can do independently

14.  Your confidence to perform Intercostal Chest Drain insertion (BEFORE attending the workshop)

a.     Afraid of doing

b.     Not sure

c.      Can do under supervision

d.     Can do independently

15.  Your confidence to perform Intercostal Chest Drain insertion (AFTER attending the workshop)

a.     Afraid of doing

b.     Not sure

c.      Can do under supervision

d.     Can do independently

16.  Your confidence to perform pericardiocentesis (BEFORE attending the workshop)

a.     Afraid of doing

b.     Not sure

c.      Can do under supervision

d.     Can do independently

17.  Your confidence to perform pericardiocentesis (AFTER attending the workshop)

a.     Afraid of doing

b.     Not sure

c.      Can do under supervision

d.     Can do independently

18.  Your confidence to perform central line insertion (BEFORE attending the workshop)

a.     Afraid of doing

b.     Not sure

c.      Can do under supervision

d.     Can do independently

19.  Your confidence to perform central line insertion (AFTER attending the workshop)

a.     Afraid of doing

b.     Not sure

c.      Can do under supervision

d.     Can do independently

20.  Your confidence to perform External Ventricular Drain insertion (BEFORE attending the workshop)

a.     Afraid of doing

b.     Not sure

c.      Can do under supervision

d.     Can do independently

21.  Your confidence to perform External Ventricular Drain insertion (AFTER attending the workshop)

a.     Afraid of doing

b.     Not sure

c.      Can do under supervision

d.     Can do independently

22.  What did you like best about the workshop?

a.     Cadavers

b.     Faculty

c.      Content

d.     Hands-on

23.  Will you recommend this workshop to other learners in the future?

a.     Not at all

b.     Cannot say

c.      Definitely

## References

[1] Sahoo SK, Gupta SK, Salunke P (2022). Setting up a neurosurgical skills laboratory and designing simulation courses to augment resident training program. Neurol India.

[2] Donoho DA, Pangal DJ, Kugener G (2021). Improved surgeon performance following cadaveric simulation of internal carotid artery injury during endoscopic endonasal surgery: training outcomes of a nationwide prospective educational intervention. J Neurosurg.

[3] Selcuk İ, Tatar I, Huri E (2019). Cadaveric anatomy and dissection in surgical training. Turk J Obstet Gynecol.

[4] Hughes JL, Katsogridakis E, Junaid-Siddiqi A, Pollard JS, Bedford JD, Coe PD, Jones DJ (2019). The development of cadaveric simulation for core surgical trainees. Bull R Coll Surg Engl.

[5] Selçuk I, Barut Ç, Çalişkan E (2019). Impact of a gynecologic oncology cadaveric dissection course for surgical training. Anatomy.

[6] Suzuki T, Suzuki-Narita M, Kubota K, Mori C (2022). Updates on cadaver surgical training in Japan: a systematic facility at Chiba University. Anat Sci Int.

[7] Shichinohe T, Kobayashi E (2022). Cadaver surgical training in Japan: its past, present, and ideal future perspectives. Surg Today.

[8] Kumar VD, Rajasekhar SS (2021). Multiple facets of learning a skill - amalgamation of learning theories in cadaveric surgical skill lab. J Adv Med Educ Prof.

[9] Lee J, Park HS, Lee DW (2021). From cadaveric and animal studies to the clinical reality of robotic mastectomy: a feasibility report of training program. Sci Rep.

[10] Henry S, Brasel K, Stewart RM (2018). ATLS Advanced Trauma Life Support Student Course Manual.

[11] Grabo D, Bardes J, Sharon M, Borgstrom D (2020). Initial report on the impact of a perfused fresh cadaver training program in general surgery resident trauma education. Am J Surg.

[12] Ghosh A, Chaudhury S (2021). Emergency cricothyroidotomy procedure: lecture on surgical anatomy and briefing of the procedure followed by skill assessment in first-semester medical students. Rev Arg de Anat Clin.

[13] Shaw MR, Hughes KE (2020). High risk, low volume: evaluation of a reusable cricothyrotomy model in a paramedic difficult airway training course. Air Med J.

[14] Kondrashova T, Canaan R, Gunn B, Pazdernik V, Houser JJ (2020). Development of competency in needle-guided procedures through the use of soft-embalmed cadavers. Mo Med.

[15] Meyer-Pflug AR, Rasslan R, Yassushi Ussami E (2022). Which model is better to teach how to perform tube thoracostomy: synthetic, cadaver, or animal?. J Surg Res.

[16] Saracoglu KT, Yilmaz M, Turan AZ, Kus A, Colak T, Saracoglu A (2020). Pecha Kucha with part-task training improves airway management in fresh frozen cadavers: a case-control observational study. Med Princ Pract.

[17] Theroux L, Steere M, Katz E, Jewell R, Gardner A (2022). A goat cadaver as a cost-effective resource for teaching emergency medicine procedures in Kijabe, Kenya. Pediatr Emerg Care.

[18] Hsu CC, Tsai SH, Tsai PJ (2022). An adapted hybrid model for hands-on practice on disaster and military medicine education in undergraduate medical students during the COVID-19 pandemic. J Acute Med.

[19] Beaven A, Griffin D, James H (2021). Highly realistic cadaveric trauma simulation of the multiply injured battlefield casualty: an international, multidisciplinary exercise in far-forward surgical management. Injury.

[20] Mantica G, Leonardi R, Diaz R (2022). Reporting ChAracteristics of cadaver training and sUrgical studies: the CACTUS guidelines. Int J Surg.

